# Anterior transcorporeal approach combined with posterior translaminar approach in percutaneous endoscopic cervical discectomy for two-segment cervical disc herniation treatment: a technical report and early follow-up

**DOI:** 10.1186/s13018-023-04471-4

**Published:** 2024-01-03

**Authors:** Zheng-Ji Wang, Qian Du, Shu-Fa Wang, Heng Su, Wen He, Wen-Bo Liao, Zhi-Jun Xin, Wei-Jun Kong

**Affiliations:** 1 Orthopedics, The Second Affiliated Hospital of Zunyi Medical University, Zunyi, Guizhou China; 2 Department of Spinal Surgery, The Affiliated Hospital of Zunyi Medical University, Zunyi, Guizhou China; 3 The Collaborative Innovation Center of Tissue Damage Repair and Regeneration Medicine of Zunyi Medical University, Zunyi, Guizhou China

**Keywords:** Two-segment cervical disc herniation, Full endoscopic technique, Minimally invasive surgery, Combination approach with full endoscopy

## Abstract

**Objective:**

Full endoscopic techniques are being gradually introduced from single-segment cervical disc herniation surgery to two-segment cervical disc herniation surgery. However, there is no suitable full endoscopic treatment for mixed-type two-segment cervical disc herniation (MTCDH) in which one segment herniates in front of the spinal cord and the other segment herniates behind the spinal cord. Therefore, we introduce a new full endoscopic technique by combining an anterior transcorporeal approach and a posterior translaminar approach. In addition, we provide a brief description of its safety, efficacy, feasibility, and surgical points.

**Methods:**

Thirty patients with MTCDH were given full endoscopic surgical treatment by a combined transcorporeal and transforaminal approach and were followed up for at least 12 months.

**Results:**

Clinical assessment scales showed that the patient’s symptoms and pain were significantly reduced postoperatively. Imaging results showed bony repair of the surgically induced bone defect and the cervical Cobb angle was increased. No serious complications occurred.

**Conclusion:**

This technique enables minimally invasive surgery to relieve the compression of the spinal cord by MTCDH. It avoids the fusion of the vertebral body for internal fixation, preserves the vertebral motion segments, avoids medical destruction of the cervical disc to the greatest extent possible, and expands the scope of adaptation of full endoscopic technology in cervical surgery.

## Introduction

Open surgery, including anterior cervical discectomy and fusion (ACDF), anterior cervical corpectomy and fusion (ACCF), and cervical disc replacement, is currently the main treatment procedure for two-segment cervical disc herniation [1, 2]. However, traditional open surgery is traumatic and may cause a series of postoperative complications, such as oesophageal injury, tracheal injury, hoarseness, vascular injury, dysphagia, endophyte displacement, and adjacent segmental disease, which seriously affect the patient’s postoperative quality of life [3–5]. Percutaneous endoscopic cervical discectomy (PECD) uses a minimally invasive approach to decompress the herniated cervical disc while providing excellent surgical results and is an ideal alternative to traditional open surgery [6].

Anterior transdiscal percutaneous endoscopic cervical discectomy (ATd-PECD) is performed by drilling a hole in the intervertebral disc to create an endoscopic channel for delivering endoscopic instruments to the site of herniation for decompression, allowing for decompression of two-segment cervical disc herniations where the upper and lower segments both herniate anterior to the spinal cord [7–9]. However, the surgical destruction of the disc by drilling can result in the loss of intervertebral height at the operated segment in the distant future [10, 11]. The amount of intervertebral height loss for the treatment of two-segment cervical disc herniation with ATd-PECD is inevitably greater than in single-segment cervical disc herniation, which also greatly limits the use of ATd-PECD in the treatment of two-segment cervical disc herniation.

Scoville [12] first proposed posterior cervical foraminotomy in 1945, avoiding interference with the intervertebral space. Adamson et al. [13] pioneered by introducing microscopic techniques into posterior cervical foraminotomy. Based on this, Ruetten et al. [14, 15] in 2007 replaced the microscope system with a full endoscopic system and introduced posterior percutaneous endoscopic cervical discectomy (P-PECD). Wagner et al. [16] first attempted to use P-PECD for continuous two-segment cervical disc herniation and obtained a better clinical result. However, in the subsequent study, P-PECD was found to be suitable only for lateral or lateral-posterior two-segment cervical disc herniations where both the upper and lower segments were on the same side and did not allow decompression of the herniation of the ventral aspect of the spinal cord [17, 18].

George et al. [19] first proposed an anterior transcorporeal discectomy in 1993, reconciling the protection of the cervical disc and the decompression of the ventral aspect of the spinal cord. Subsequently, different surgeons have made a series of improvements on this basis, but all of these improved procedures are also performed under microscopic or open surgical conditions [20–25]. Deng [26] was the first to propose anterior transcorporeal percutaneous endoscopic cervical discectomy (ATc-PECD) after combined anterior transcorporeal discectomy with a full endoscopic technique in 2016. However, since the transcorporeal approach endoscopic technique emerged late, resulting in the technique not being sufficiently mature, its use in the treatment of two-segment cervical disc herniation has not been reported.

**Fig. 1:**
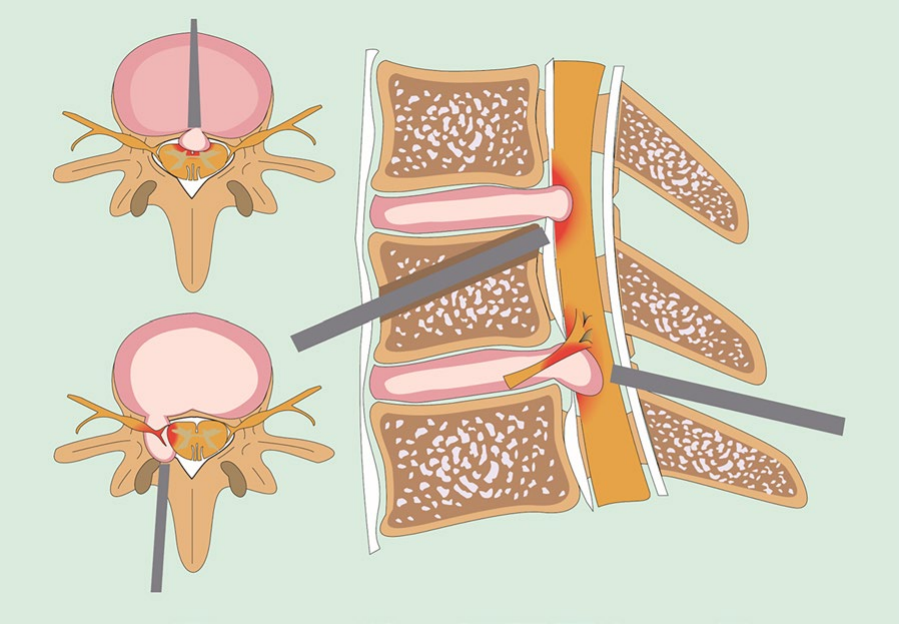
The endoscopic technique diagram that combines anterior transcorporeal and posterior transforaminal approaches

In previous studies of full endoscopic treatment of cervical disc herniations, there are already proven treatment strategies for two-segment cervical disc herniations in which both upper and lower segments herniate at the anterior or lateral-posterior aspect of the spinal cord. However, there is no effective full endoscopic treatment measure for MTCDH in which one segment herniates at the front of the spinal cord and the other segment herniates at the lateral-posterior aspect of the spinal cord. Through description of a typical case, we present a new full endoscopic technique that combines anterior transcorporeal and posterior transforaminal approaches for the handling of this condition (Fig. 1). We aimed to achieve minimally invasive decompression for mixed two-segment cervical disc herniation, avoid fusion internal fixation of the vertebral body, and reduce damage to the normal disc.

## Methods

### Patient selection

This study received approval from the local ethics committee, and all patients provided informed consent. The inclusion criteria were as follows: (1) the patient had MTCDH in which one segment herniated in the anterior part of the spinal cord and the other segment herniated in the lateral-posterior aspect of the spinal cord; (2) patients who are ineffective on conservative treatment; (3) preoperative imaging results were consistent with the patient’s neurological symptoms; and (4) patients with mild spinal cervical spondylosis at or below Nurick III level. The exclusive criteria were as follows: (1) patients with single-segment and more than two-segment cervical disc herniation; (2) patients with two-segment cervical disc herniation of the same type; (3) patients with cervical instability; (4) patients with coagulation disorders; and (5) patients with a history of cervical spine surgery. Based on the above criteria, a total of 30 patients were enrolled in the study during 2019–2021. The demographics and clinical characteristics of the patients are presented in Table 1.

**Table 1:** Summary of patient demographics and clinical characteristics

Basic features	Value
Age (years)	51.17 ± 9.01 (35–67)
Genders (male/female)	17/13
BMI (kg/m^2^)	24.49 ± 2.06 (21.3–28)
Smoking	14 (46.7%)
Duration of symptoms (weeks)	13.1 ± 3.92 (6–21)
Continuous double segment	24(80.0%)
Hoffman positive	9 (30%)
Knee hyperreflexia	12 (40%)
Neck pain	28(93.3%)
Numbness and weakness in hands	30 (100%)

Values are number of patients (percentage) or mean ± SD (range)

### Case presentation

**Fig. 2:**
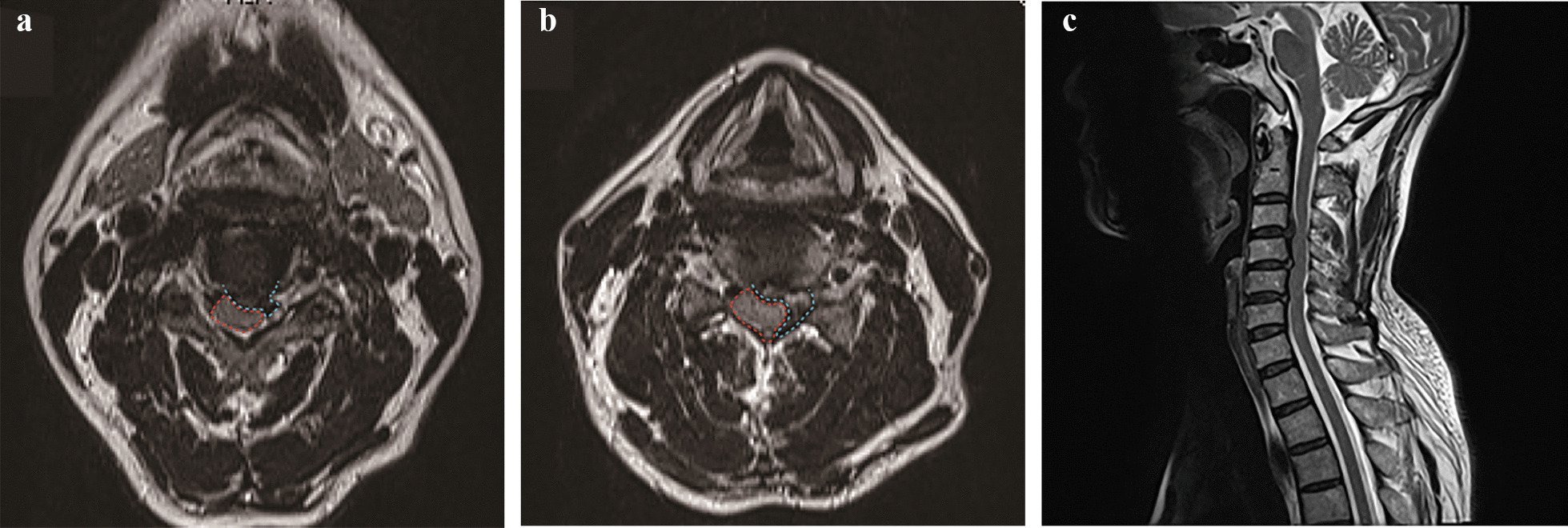
Preoperative MRI images of the cervical region of the patient. **a** T1-weighted axial view showing the herniation of the disc (blue line) to the anterior aspect of the spinal cord (red line) at the C3/4 level; **b** T1-weighted axial view showing the herniation of the disc (blue line) to the lateral-posterior aspect of the spinal cord (red line) at the C4/5 level; and **c** T2-weighted sagittal view showing the spinal cord of the patient being compressed by the herniated disc

**Fig. 3:**
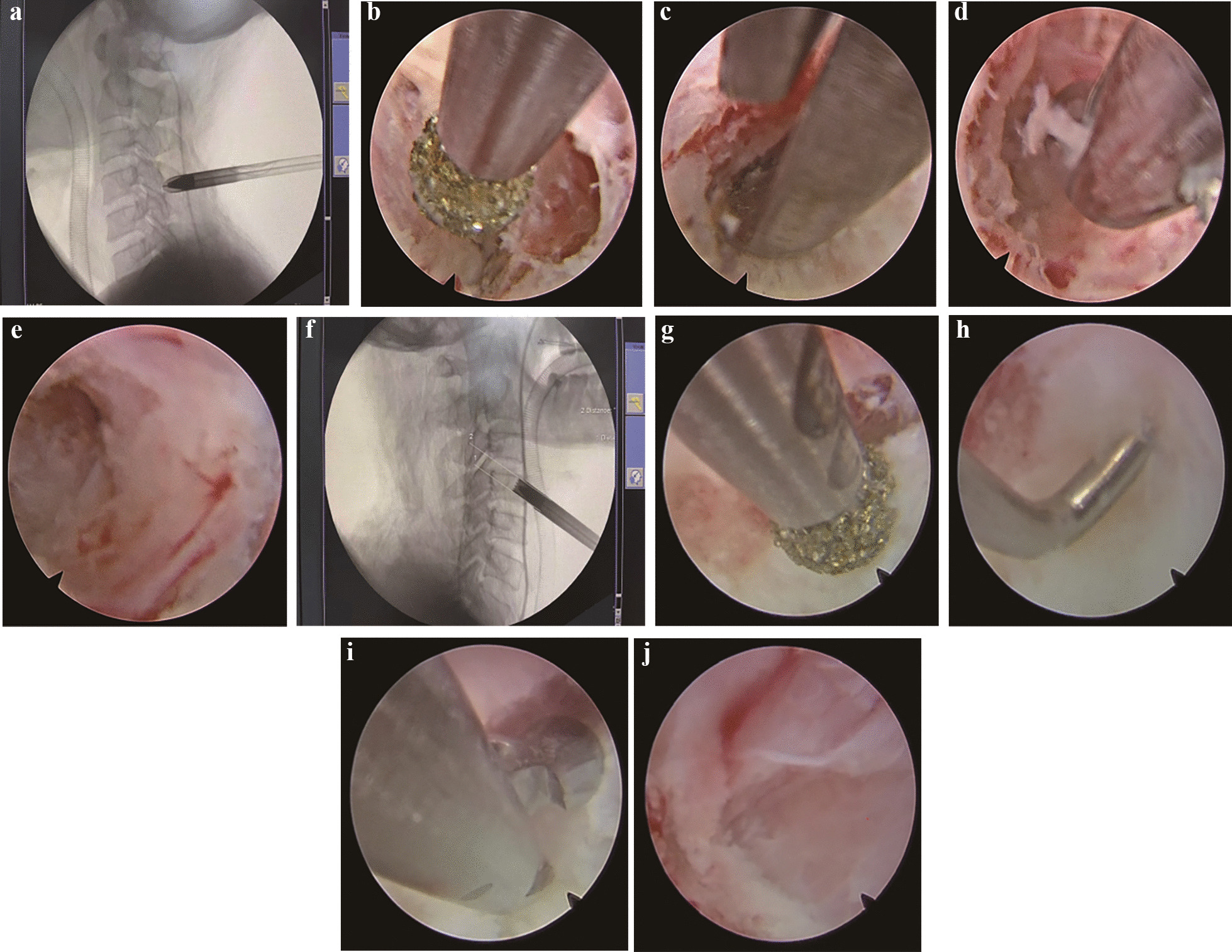
Intraoperative C-arm scan and endoscopic images. **a** Intraoperative C-arm sagittal fluoroscopy showing the placement of the spreader along the kyphotic needle to the “V” point. **b** A high-speed diamond drill is used to thin the lamina. **c** The remaining thin bone fragments are removed with occlusal forceps. **d** The herniated disc tissue is removed with nucleus pulposus forceps. **e** The spinal cord and nerve roots are sufficiently decompressed. **f** Intraoperative C-arm sagittal fluoroscopy shows the use of a trephine to create a transcorporeal bone channel. **g** The residual bone in the bone channel is polished using a diamond high-speed drill. **h** Use of a blunt hook to confirm the opening of the posterior wall of the bone channel. **i** Removal of the herniated disc tissue using nucleus pulposus forceps. **j** The dural sac notably re-expands after herniated disc removal

**Fig. 4:**
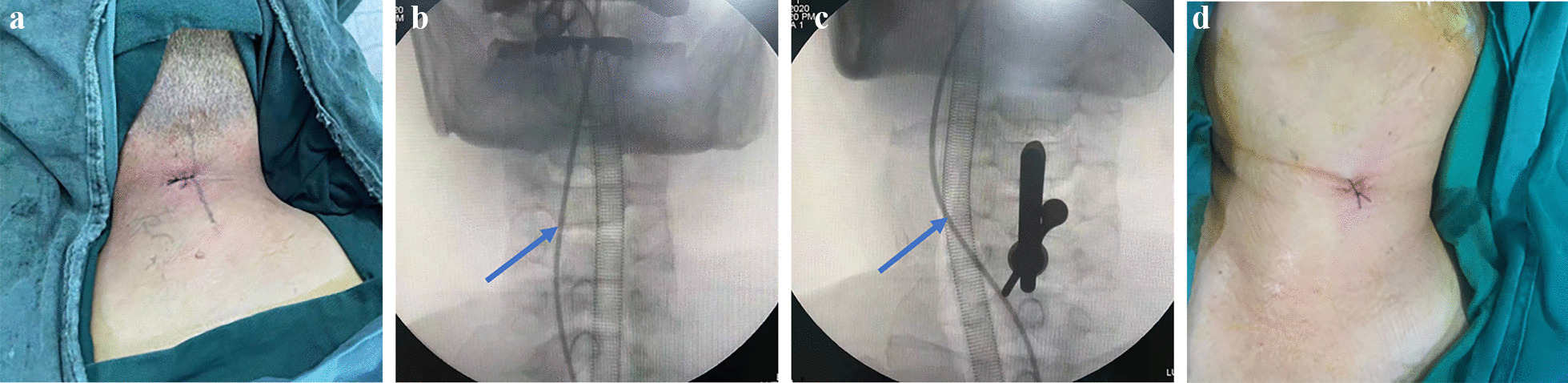
Intraoperative C-arm scan and postoperative wound pictures. **a** The wound of after P-PECD. **b**, **c** Intraoperative C-arm fluoroscopy showing the position of the oesophagus marked by iohexol in the gastric tube (blue line). **d** The wound of after ATc-PECD

**Fig. 5:**
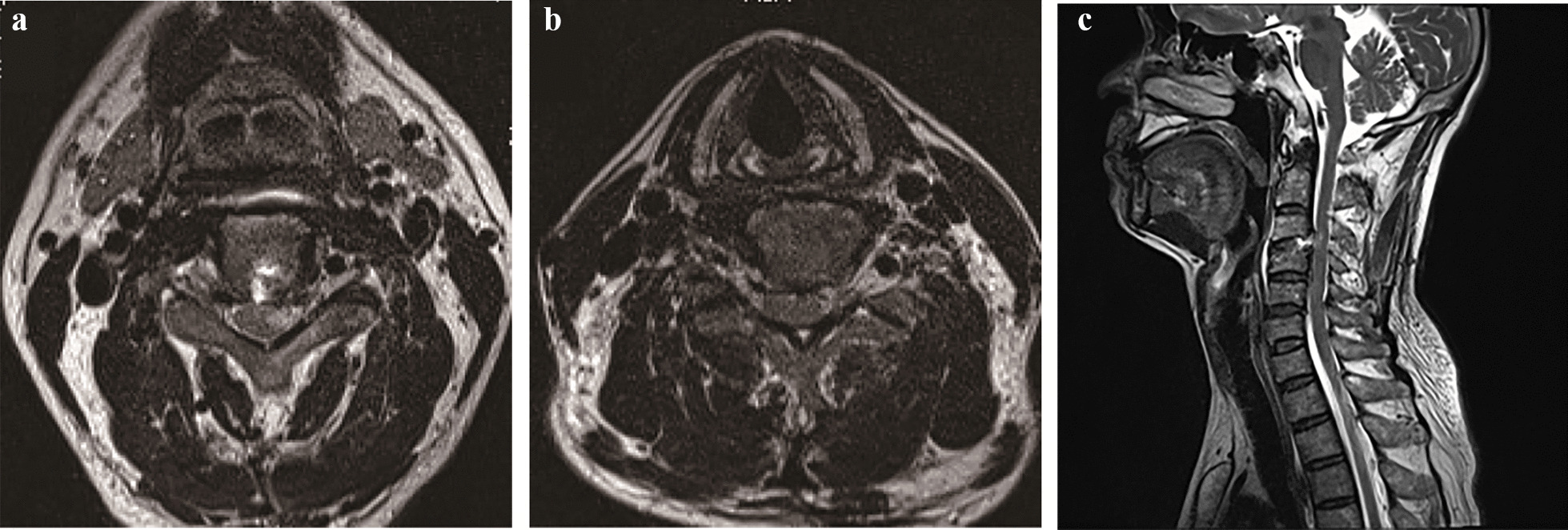
MRI images of the patient 1 week after surgery. **a**, **b** T1-weighted axial view shows that the discs compressing the spinal cord at the C3/4 and C4/5 levels were extracted. **c** T2-weighted sagittal view shows that the patient’s spinal canal is patent and the spinal cord compression has been released

**Fig. 6:**
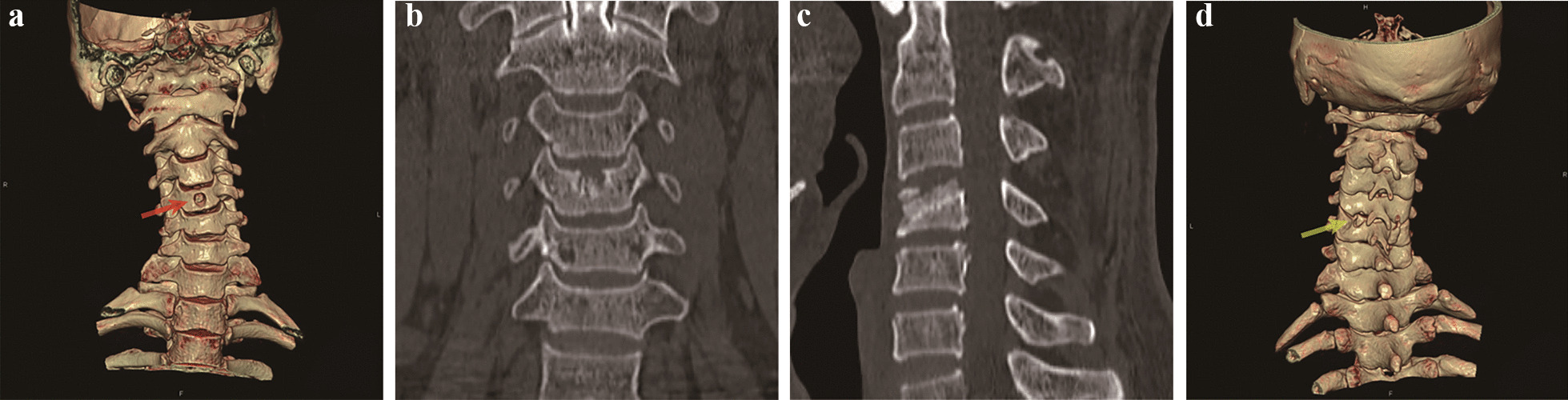
CT images of the patient 1 week after surgery. **a** CT 3D reconstruction showing the location of the vertebral bone channel entrance (red arrow). **b** Coronal view showing the C4 vertebral body bone channel and the internal autogenous bone graft. **c** Sagittal view showing the C4 vertebral bone channel and the internal autologous bone graft. **d** CT 3D reconstruction showing the entrance to the C5 lamina bone window (yellow arrow)

**Fig. 7:**
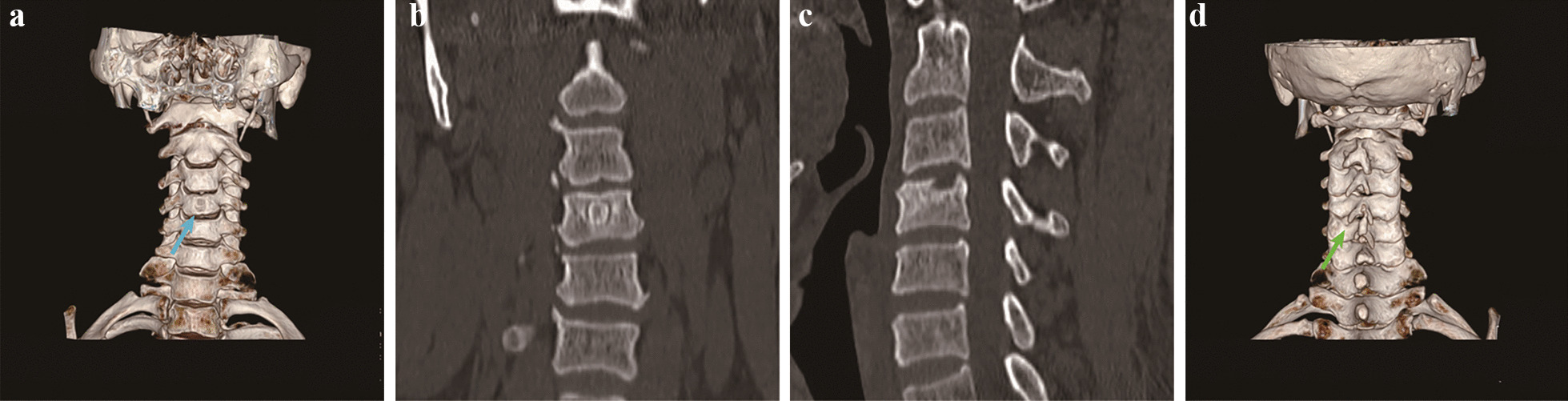
CT images of the patient 6 months after surgery. **a** CT 3D reconstruction showing the location of the residual entrance to the C4 vertebral channel (blue arrow). **b** Coronal view showing that the C4 vertebral bone channel is healed and the vertebral body is not collapsed. **c** Sagittal view showing that the C4 vertebral body bone channel is healed. **d** CT 3D reconstruction showing that the C5 vertebral plate bone window is healed (green arrow)

A 56-year-old man presented with left neck and shoulder pain for 12 + weeks and limited range of motion. Conservative treatment was ineffective. Neurological examination of the left upper extremity revealed that it was markedly numb with decreased biceps muscle strength (grade 3), and both upper extremities were positive for the Hoffmann sign, with a visual analogue scale (VAS) of 7 and a Japanese Orthopaedic Association (JOA) score of 11. Magnetic resonance imaging (MRI) showed that the cervical disc in the C3/4 segment protruded into the anterior aspect of the spinal cord, and the cervical disc in the C4/5 segment protruded into the lateral-posterior aspect of the spinal cord (Figs. 2, 3, 4, 5, 6, and 7).

### Endoscopic instruments

The spine full endoscopy system (SPINENDOS GmbH, Munich, Germany) consists of a 6.9-mm-diameter outer sheath and a 4.3-mm-diameter working channel, a high-speed grinding drill, a continuous water irrigation system, a trephine with a 7.5-mm outer diameter and a 6.5-mm inner diameter, and a low-temperature radiofrequency ablation system.

### Technique

The surgery was performed by the corresponding author. We first utilized the full endoscope to resected the herniated cervical disc at the C4/5 segment from the posterior approach. Twenty millilitres of iohexol was injected into the nasogastric tube before the start of the procedure to enable visualization of the patient’s oesophageal trajectory under C-arm radiation. The patient was placed in a prone position, the head was fixed with tape, the neck was bent, and the hands were fastened to the sides of the body. With C-arm monitoring, the Kirschner needle was inserted through the skin to the “V” point at the junction of the upper and lower articular processes, a 7-mm-diameter incision was made in the skin centred on the puncture point, and the spreader was placed along the Kirschner needle to the “V” point through the incision (Fig. 3a); then, the working cannula was placed along the spreader, the spreader was removed, and the endoscopic instrument inserted from the working cannula. The surgery was performed with a continuous flush of 0.9% saline, and the saline bag was placed at a height of approximately 1 m from the operating table. To avoid damage to the nerve roots and spinal cord, we first used a high-speed diamond grinding drill to thin the lamina along the “V” point (Fig. 3b), then used bone biting forceps to remove the remaining thin bone, forming a window of approximately 7 mm in diameter (Fig. 3c) to reveal the compression area, and used medullary forceps to remove the protruding disc (Fig. 3d). The operation process must be gentle and should not overstrain the spinal cord and nerve roots. After completion of decompression (Fig. 3e), the endoscopic system was removed, active bleeding was observed during withdrawal, the skin was stitched up (Fig. 4a), and the wound was covered with sterile gauze without placing drains.

The patient was turned and repositioned to be supine, the patient’s head was secured with tape, and the patient’s neck was extended. Compared to the traditional transdiscal endoscopic approach, we planned to create a direct transcorporeal endoscopic approach to the herniated disc in the C4 vertebral body. C-arm fluoroscopy was used to visualize the position of the oesophagus and trachea (Fig. 4b). The surgeon used the two-finger method to isolate a safe gap between the left carotid sheath and the visceral sheath (the surgeon touched the patient’s carotid artery pulsation with the index finger and pushed the artery outward, while the middle finger was used to push the oesophageal trachea inward) (Fig. 4c). With C-arm monitoring, the Kirschner needle was inserted through this gap to the anterior lower part of the C4 vertebral body, a 7-mm incision was made around the Kirschner needle puncture point, a spreader was used to open the soft tissue channel along the Kirschner needle, and a working cannula was placed along the spreader to establish an endoscopic soft tissue channel. The spreader was removed, and the trephine was inserted (Fig. 3f). The trephine was used to drill the vertebral body with monitoring through the C-arm. When the trephine reached the posterior superior edge of the C4 vertebral body, the trephine was lightly shaken to remove the central bone strip. The trephine was withdrawn and placed alongside the endoscopic instruments, and a diamond high-speed grinding drill was used to polish the remaining bone of the channel (Fig. 3g). When the channel was established, a blunt hook was used to probe the posterior wall of the channel for penetration (Fig. 3h), followed by the use of pulpal forceps to remove the herniated disc (Fig. 3i). Finally, the posterior longitudinal ligament was broken open, and the return of dural sac expansion was observed as indicating the completion of decompression (Fig. 3j). Endoscopic instruments were withdrawn, and the site was checked for active bleeding; the previously removed bone strips were trimmed, and the bone strips were replanted. The endoscopic instruments were withdrawn without placing a drain, and the wound was sutured and covered with sterile gauze (Fig. 4d).

### Follow-up and efficacy evaluation

Surgical complications (dural injury, spinal cord injury, hematoma, oesophageal vascular injury, wound infection, etc.), symptomatic relief, operative time, and postoperative hospitalization were recorded. At each follow-up visit, the VAS was used to assess the patient’s pain, and the JOA score was used to assess the patient’s neurological symptoms, which was compared with the patient’s preoperative condition. At the final follow-up, patient recovery was assessed using modified MacNab criteria.

MRI was used to assess the effectiveness of surgical decompression, and computed tomography (CT) was used to assess the healing of the vertebral bony channels. Cervical spine lateral X-ray films was used to assess the patient’s cervical spine Cobb angle. Cervical stability of surgical segments assessed using hyperextension and hyperflexion radiographs.

### Statistical analysis

The statistical analysis was conducted using the Statistical Package for Social Sciences (SPSS version 22.0, SPSS Inc., Chicago, IL). The paired samples t-test was employed for data comparisons. Statistical significance was defined as a *P*-value ≤ 0.05. The results are presented as mean ± standard deviation (SD).

## Results

### Clinical outcomes

All 30 patients included in this study were successfully underwent surgery, and the patients’ symptoms improved significantly after surgery. The average operative time was 120.70 ± 9.07 (range, 105–134) minutes. The average hospitalization time was 61.83 ± 5.03 (range, 52–72) hours. Patients were advised to wear a neck brace for a minimum of 3 weeks, and regular follow-up visits were conducted over a 12-month period to assess the clinical outcomes. The JOA score indicated that the patient’s neurological function was restored, and the VAS indicated that the patient’s pain was significantly relieved (Table 2). The modified MacNab criteria score at final follow-up showed that the excellent and good rates were 86.7%.

**Table 2:** Follow-up records of VAS, JOA scores, and C2–C7 cervical Cobb angle during the follow-up period

	Pre	1 month	3 months	6 months	12 months
*VAS*					
Neck	6.53 ± 1.59	3.70 ± 1.37*	2.17 ± 1.09*^∆^	1.27 ± 0.98*^∆◊^	0.87 ± 0.68*^∆◊#^
Hands	5.37 ± 1.27	2.80 ± 1.27*	1.97 ± 0.89*^∆^	1.43 ± 0.89*^∆◊^	0.67 ± 0.61*^∆◊#^
JOA	8.80 ± 1.99	12.50 ± 1.50*	13.53 ± 0.78*^∆^	14.10 ± 0.76*^∆◊^	14.40 ± 0.67*^∆◊#^
Cobb angle (°)	10.58 ± 1.68	11.34 ± 1.44*	11.56 ± 1.39*^∆^	11.83 ± 1.41*^∆◊^	11.98 ± 1.31*^∆◊^

Values are mean ± SD

*Compared with preoperative value, *P* < 0.05; ∆compared with postoperative value at 1 month, *P* < 0.05; ◊compared with postoperative value at 3 months, *P* < 0.05; #compared with postoperative value at 6 months, *P* < 0.05

### Radiological outcomes

All patients underwent preoperative examinations including X-ray, CT, and MRI. Follow-up examinations were conducted at 1 week postoperatively using the same imaging modalities. Twenty-eight patients underwent postoperative X-ray examinations at 1, 3, 6, and 12 months postoperatively. Twenty-five patients underwent postoperative CT examinations at 6 months postoperatively. The MRI results showed that all herniations were removed (Fig. 5). At 6 months postoperatively, the CT results showed no endplate collapse or vertebral fracture in any of the operated segments, and the results also indicated that the bony channels in the vertebral body and the bony windows on the vertebral plate had been significantly repaired (Figs. 6, 7). Dynamic X-ray results showed no cervical instability occurred in the operated segments, and static X-rays indicated that the C2–C7 cervical Cobb angle was increased (Table 2).

### Complications

All patients had a mild postoperative cervical oedema, which naturally subsided 3–6 h after surgery. Two patients had blood leakage from the wound during hospitalization, which ceased after compression was applied. There were no other intraoperative or postoperative complications such as dyspnoea, dysphagia, hoarseness, arterial injury, oesophageal injury, spinal cord injury, cerebrospinal fluid leakage, or infection.

## Discussion

At present, in the utilization of full endoscopic technology for the treatment of two-segment cervical disc herniation, all decompression is accomplished in one segment and then reversed to complete the decompression in the other segment, which requires the direction of the disc herniation to be consistent [18]. However, in realistic situations, cervical disc herniation of both the upper and lower segments is mostly likely to be in two opposite directions, the anterior and posterior; such MTCDHs are not amenable to decompression by a single-entry full endoscopic surgical technique. Therefore, we introduce a new combined-approach full endoscopic technique for handling this condition and demonstrate a positive treatment effect in preliminary clinical trials. Contrary to the previous single-entry full endoscopic technique, which could only be used to treat continuous two-segment cervical disc herniations, the combined-approach full endoscopic technique allow decompression of noncontinuous cervical disc herniations, affording greater flexibility.

There are two clinical approaches for full endoscopic decompression at the ventral aspect of the spinal cord: the transcorporeal approach or the transdiscal approach. In this study, we chose the transcorporeal approach rather than the transdiscal approach. There are two reasons for this: First, the transcorporeal approach is more extensive in the area of decompression than the transdiscal approach. Although the transdiscal approach is effective for herniation decompression in the disc plane, due to obstruction of the vertebral body, the transdiscal approach is not effective in posterior free herniation of the vertebral body. The transcorporeal approach allows the bone channel to be constructed according to preoperative imaging data, and the decompression plane can be flexibly adjusted between the vertebral and disc planes [27–29]. Furthermore, low intervertebral spaces, calcified discs, and large bones in the intervertebral space will prevent the operation of the transdiscal approach [30]. In contrast, the transcorporeal approach does not present these concerns. Second, the transcorporeal approach can reduce the disturbance to the intervertebral space during surgery [26, 31, 32]. The endoscopic instrumentation in the transdiscal approach must pass through the anterior normal disc tissue before reaching the herniated area, and this process will inevitably cause damage to the anterior normal disc. Drilling damage to the intervertebral disc is an important cause for the distant loss of intervertebral height in total endoscopic surgery [10, 11]. In contrast, the transcorporeal approach avoids damage to the disc by creating a bony channel in the vertebral body, bypassing the normal disc tissue at the anterior aspect. Ren et al. [33] also confirmed in a comparative study of ATc-PECD and ATd-PECD that the decrease in intervertebral height was less with a transcorporeal approach than with a transdiscal approach.

In previous studies, due to the complex and diverse expression of two-segment cervical disc herniation, it was difficult to completely decompress the disc regardless of full endoscopic surgical approach, and open anterior decompression and internal fixation were often preferred [34]. However, both ACCF and ACDF will inevitably cause the destruction of the stable structures of the cervical spine, such as the vertebral body and the intervertebral disc, and although the stability of the cervical spine can be reconstructed by using bone graft fusion and plate internal fixation, this is achieved at the expense of the mobility of the cervical spine [35, 36]. In this study, the two approaches we chose did not interfere with the intervertebral space, avoiding unnecessary damage to the segmental disc operated on; although damage to the vertebral body was unavoidable, the integrity of the vertebral body suffered no serious damage, and we observed that when there was surgical damage to both the vertebral body and the lamina, the bone-forming effect could be relied on to complete the repair. When we performed ATc-PECD on the C3/4 disc, unlike previous studies that involved using diamond high-speed grinding drills to create the transcorporeal bone channel, our approach involved used a trephine instead. This enabled the operator to individualize the optimal diameter of the bone channel according to the size of the vertebral body and disc, avoiding excessive grinding of the vertebral body while allowing the resected central bone strip to be replanted and used to promote the repair of the vertebral bone channel, which served further to maintain the integrity of the vertebral body. In our previous study of single-segment cervical disc herniation, we demonstrated that replanting the central bone strip can promote healing of the bone channel, and no patients experienced cervical instability [28]. The zygapophysial joint is an important structure for maintaining the stability of the cervical spine posteriorly, but during posterior cervical foraminotomy decompression, to completely expose the nerve roots, a portion of the zygapophysial joint has to be ground away. Zdeblick et al. [37], in a cadaveric study, found that grinding more than 1/2 of the zygapophysial joint would cause a serious disruption of cervical stability; in contrast, grinding less than 1/2 did not cause significant cervical instability. This finding was also confirmed in our previous study [38]. We also chose to preserve more than 1/2 of the zygapophysial joint when performing P-PECD on the C4/5 disc. At the follow-up, no significant changes in cervical stability were observed, and the imaging data showed that the bone window on the foramina achieved bony healing. Based on the good results of ATc-PECD and P-PECD in single-segment cervical disc herniation treatment, we combined the above two endoscopic approaches in the surgical treatment of MTCDH and successfully preserved the motion segment of the patient’s cervical spine.

The soft tissues of the cervical region are rich in nerves and blood vessels, and the inappropriate pulling of the soft tissues of the cervical region or the misuse of surgery may cause damage to the blood vessels and nerves, which could lead to corresponding complications. In the anterior transcorporeal full endoscopic approach, we use the “two-finger method” to separate the arterial sheath and visceral sheath so that the endoscopic instruments could reach the vertebral body through this natural tissue gap, and all operations are performed under the working sleeve. Strain to the cervical soft tissues and direct contact between the endoscopic instruments are avoided, which can reduce the risk factors for complications related to soft tissue injury in anterior cervical surgery [26]. The posterior cervical approach has unparalleled advantages for lateral cervical disc herniation extraction because there are no important anatomical structures in the posterior cervical tissues, the surgical instruments can reach the spinal canal directly through the lamina space, and the risk is low. However, the spinal canal is isolated from the skin by hypertrophic muscles, and open posterior cervical foraminotomy requires extensive stripping of the posterior cervical muscles. Injury of the musculoligamentous complex is one of the important causes of axial symptoms in the patient’s postoperative neck [39]. The full endoscopic technique only requires a small incision for decompression, without further extensive dissection of the posterior cervical muscles, reducing the possibility of posterior cervical axial symptoms [15]. The placement of exogenous endografts alters the inherent biomechanical structure of the cervical spine, which is an important risk factor for adjacent segmental degeneration [40, 41]. Studies have shown that the incidence of adjacent segmental disease is approximately 12–25% after cervical fusion, and the reoperation rate is approximately 6–12% [42–45]. Through the full endoscopic technique, there is no need for further fusion and internal fixation of the vertebral body; thus, large changes in the biomechanical structure of the patient’s neck do not occur, and adjacent segmental disease due to the placement of internal implants is avoided.

Although we obtained satisfactory results in the preliminary clinical trial, there are still some limitations of this study. The full endoscopic technique reduces the damage to the cervical soft tissues, it does not completely avoid the possibility of complications related to soft tissue injury; instead, patients who accept this technique bear the risk from both anterior and posterior cervical complications. And this technique avoids the devastating damage to the discs and annulus fibrosus, but minor impairment is unavoidable. In addition, due to the steep learning curve of this technology, and the high level of surgical skill required of the surgeon, it may make the dissemination of this technology difficult. Surgeons should be alert to the threat of intraoperative radiation exposure. Studies have shown that surgeons in full endoscopic surgery are exposed to far more radiation than in open surgery, and that surgeons performing 291 unprotected full endoscopic procedures per year is the limit, while effective protection can substantially exceed this limit [46]. Computer-assisted navigation technology is expected to provide new strategies to minimize surgeon radiation exposure during full endoscopic procedures and more secure surgical options [47]. Finally, as a result of the number of cases being too small and the short follow-up period, the efficacy of this technique needs to be further investigated. Therefore, conducting further experiments with larger sample sizes and longer observational periods will be key as we continue our research with a view to minimizing complications and enhancing efficacy.

## Conclusions

We describe a new full endoscopic technique that combines the anterior transcorporeal approach and the posterior transforaminal approach in the treatment of two-segment cervical disc herniation. In addition, we demonstrate the feasibility and effectiveness of using full endoscopy to extract MTCDHs in which the upper and lower segments herniate at the anterior aspect of the spinal cord and the lateral-posterior aspect of the spinal cord, respectively. The technique reduces risk factors related to the disc, the vertebral body, and surgery-related complications while preserving the cervical motion segment. This is another full endoscopic technique breakthrough in the treatment of two-segment cervical disc herniation.

## Data Availability

The datasets used during the current study are available from the corresponding author on reasonable request.

## Abbreviations

ACDF: Anterior cervical discectomy and fusion
ACCF: Anterior cervical corpectomy and fusion
MTCDH: Mixed-type two-segment cervical disc herniation
PECD: Percutaneous endoscopic cervical discectomy
ATd-PECD: Anterior transdiscal percutaneous endoscopic cervical discectomy
P-PECD: Posterior percutaneous endoscopic cervical discectomy
ATc-PECD: Anterior transcorporeal percutaneous endoscopic cervical discectomy
JOA: Japanese Orthopaedic Association
VAS: Visual analogue scale
CT: Computed tomography
MRI: Magnetic resonance imaging
